# Flavonoids and phenolic compounds: a promising avenue for neurodegenerative disease therapy

**DOI:** 10.55730/1300-0152.2767

**Published:** 2025-10-10

**Authors:** Teslime Özge ŞAHİN, Özge CEMALİ, Merve ÖZDEMİR, Şerife AYTEN, Duygu AĞAGÜNDÜZ

**Affiliations:** 1 Department of Nutrition and Dietetics, Faculty of Health Sciences, Afyonkarahisar Health Sciences University, Afyonkarahisar, Turkiye; 2 Department of Nutrition and Dietetics, Faculty of Health Sciences, Trakya University, Edirne, Turkiye; 3 Center for Molecular Prediction of Inflammatory Bowel Disease, Clinical Medicine, Aalborg University, Copenhagen, Denmark; 4 Department of Nutrition and Dietetics, Faculty of Health Sciences, Gazi University, Ankara, Turkiye

**Keywords:** Antioxidants, neuroinflammation, neuroprotection, phytotherapy

## Abstract

**Background/aim:**

Neurodegenerative diseases such as Alzheimer’s, Parkinson’s, Huntington’s, and ALS are characterized by a progressive loss of nerve cells, for which no definitive cure currently exists. These conditions share common pathological mechanisms, including chronic neuroinflammation, oxidative stress, protein aggregation, and mitochondrial dysfunction. Flavonoids and other plant-derived phenolic compounds have recently attracted attention for the treatment of such conditions due to their antiinflammatory and antioxidant properties. This review explores the neuroprotective mechanisms of flavonoids and evaluates their potential for the prevention and treatment of neurodegenerative diseases.

**Materials and methods:**

A literature search of the Web of Science, PubMed, and ScienceDirect databases was conducted to evaluate the therapeutic potential of flavonoids and phenolic compounds against neurodegenerative diseases. The search terms included “polyphenols”, “flavonoids”, and related compounds, along with “Alzheimer’s”, “Parkinson’s”, “Huntington’s”, and “Amyotrophic lateral sclerosis”. Eligible studies included clinical trials, randomized controlled trials, and in vitro and in vivo research published in English. Priority was given to studies from the last decade, although older but significant publications were also included.

**Results:**

The findings of multiple studies report the ability of flavonoid compounds such as quercetin, myricetin, apigenin, and epigallocatechin gallate (EGCG) to modulate critical signaling pathways, reduce oxidative stress, prevent the accumulation of neurotoxic proteins, and support mitochondrial function. These bioactive molecules have exhibited significant potential in slowing disease progression and preserving neuronal integrity. Their therapeutic application, however, has been limited by their poor bioavailability, low stability, and rapid metabolism.

**Conclusion:**

Flavonoids have shown promise as naturally derived agents with multi-targeted activity against neurodegenerative processes. Enhancing their absorption and stability through novel delivery systems and structural modifications could significantly improve their clinical efficacy. When administered early or as a complementary therapy, flavonoids can be considered a safe and effective approach to the management of neurodegenerative diseases.

## 1. Introduction

Neurodegenerative diseases are characterized by progressive neuron loss or dysfunction. Although hundreds of neurodegenerative diseases have been identified, research and public interest are predominantly directed toward Parkinson’s disease (PD), Alzheimer’s disease (AD), amyotrophic lateral sclerosis (ALS), and Huntington’s disease (HD) (Maher, 2019). Neuroinflammation, oxidative stress, impaired mitochondrial function, misfolded protein aggregation, and apoptotic factors play essential roles in the pathogenesis of these diseases (Solanki et al., 2015; Moujalled et al., 2021). Another common feature of these diseases is their progressive nature, which limits the treatment options targeting the alleviation of symptoms and disease progression. Consequently, the development of safe, natural, and lifelong-applicable therapeutic alternatives holds significant clinical and scientific importance. Due to their low toxicity, flavonoids represent promising and safer therapeutic alternatives to the synthetic drugs commonly used in the treatment of neurodegenerative diseases, which often come with significant adverse effects (Rębas, 2025).

Phenolic compounds, notably polyphenols, possess antioxidant and antiinflammatory properties that have been demonstrated to inhibit the aggregation of the pathological proteins implicated in several neurodegenerative diseases characterized by cognitive decline, including AD, PD, dementia with Lewy bodies, and multiple system atrophy (Caruso et al., 2022). The focus of this review, however, is flavonoids, as the largest and most common group of phenolic compounds. Flavonoids are commonly classified into six groups: flavonols, flavones, flavanones, flavan-3-ols, anthocyanidins, and isoflavones, and are commonly found in fruit and vegetables (Solanki et al., 2015).

There has been numerous epidemiological studies to date highlighting the neuroprotective potential of dietary flavonoids (Beking and Vieira, 2010; Gao et al., 2012). In one study, after adjusting for various confounding factors, males in the highest quintile of total flavonoid consumption (631 mg/day) exhibited a 40% lower risk of PD than those in the lowest quintile (105 mg/day). Further pooled analyses have shown anthocyanins and anthocyanin-rich foods, such as berries, to be significantly correlated with reduced PD risk (Gao et al., 2012).

Identifying modifiable dietary factors that can support cognitive health may have significant benefits to public health (Ran et al., 2021). The economic burden associated with the treatment of neurodegenerative diseases should be reduced, primarily through preventive strategies, and if prevention is not possible, through economically viable treatment options. This review has been conducted to identify the polyphenols, specifically flavonoids, that are linked directly to the etiology of neurodegenerative diseases and to evaluate their therapeutic potential.

## 2. Literature search strategy

A literature search was conducted to identify studies investigating the therapeutic potential of flavonoids and phenolic compounds against neurodegenerative diseases. To this end, the Web of Science, PubMed, and Science Direct databases were searched using the key words: “polyphenols”, “flavonoids”, “phenolic acids”, “anthocyanins”, “isoflavones”, “epigallocatechin gallate”, “catechins”, “gallic acid”, and “quercetin”, crossed with the terms “Alzheimer’s disease”, “Parkinson’s disease”, “Huntington’s disease”, and “Amyotrophic lateral sclerosis”.

Eligible studies included clinical trials, randomized controlled trials, and in vitro and in vivo studies in the English-language studies. While priority was given to studies published within the last 10 years, older articles that were deemed highly relevant were also considered to strengthen the discussion.

## 3. Flavonoids and phenolic compounds: definitions, classification, and natural sources

Bioactive substances are natural compounds that support human health beyond satisfying basic nutritional needs, among which, phenolic compounds—also known as polyphenols or polyhydroxyphenols—are particularly noteworthy. These compounds are found in abundance in plants, and usually have two or more phenol rings. In nature, they contribute to the shielding of plants from environmental stressors such as oxidative damage, UV rays, and dangerous microbes (Sanches-Silva et al., 2020). The two main categories of phenolic substances are flavonoids and phenolic acids, both of which feature in plant-based diets and contribute significantly to health.

The phenolic acids that are produced naturally in plants have been associated with a number of health advantages, and are often categorized into two main forms: hydroxybenzoic acids and hydroxycinnamic acids. The bran layer of cereal grain is host to the majority of its hydroxybenzoic acids, including gallic, vanillic, protocatechuic, p-hydroxybenzoic, and salicylic acids. Conversely, hydroxycinnamic acids, which are mostly found in the outer portions of cereals, include p-coumaric, ferulic, sinapic, and caffeic acids. These substances are particularly noted for their potent antioxidant properties (Banwo et al., 2021).

### 3.1. Flavonoid subclasses and structures

More than 6000 distinct flavonoid structures have been identified, making them one of the most diverse classes of phenolic chemicals. Their basic C6–C3–C6 structure consists of two aromatic rings (A and B) linked by a three-carbon bridge that forms a third, heterocyclic pyran ring (C) (Al-Khayri et al., 2022). Many flavonoids naturally occur as glycosides, in which the aglycone is attached to a sugar moiety (pyranose or furanose) via a glycosidic bond at the first carbon, affecting their bioavailability and stability (Soto-Blanco, 2022).

Dietary flavonoids are divided into several subclasses (Figure): flavanols (e.g., quercetin, kaempferol, myricetin); flavones (e.g., apigenin, luteolin); flavanones (e.g., naringenin, hesperidin); flavan-3-ols (catechins); anthocyanins (e.g., cyanidin, delphinidin); and isoflavones (e.g., genistein, daidzein) (Banwo et al., 2021).

### 3.2. Flavonoid classes and their natural sources

Flavones have a double bond between C2 and C3 and a carbonyl group at C4, but lack a hydroxyl group at C3. They are commonly found in flowers and are abundant in vegetables like celery, parsley, carrot, and artichoke. Luteolin and apigenin are the most studied members of the group due to their antioxidant, antiinflammatory, and anticarcinogenic activities (Brodowska, 2017; Hostetler et al., 2017), and are abundant in juniper berries, radicchio, sorghum, black pepper, mint, dried parsley, thyme, thistle, artichoke, celery, vine cress, and spinach (Banwo et al., 2021).

Flavanones, common in citrus fruits, lack the C2 = C3 double bond and possess a chiral center at C2. Naringenin, eriodyctiol, and hesperidin demonstrate significant antiinflammatory, antiviral, and antioxidant effects (Purushothaman and Sumathi, 2022), and are found primarily in citrus fruit and their juices (Banwo et al., 2021).

Flavonols are widely distributed in fruit and vegetables, and include quercetin (onion, apple), kaempferol (broccoli, spinach), and myricetin (berries, wine), exhibiting strong antioxidant and antiinflammatory properties due to their high hydroxyl group content (Jomova et al., 2017; Simunkova et al., 2021). These compounds can be found in a wide variety of fruit and vegetables, such as radishes, broccoli, okra, onions, peppers, dill weed, ginger, cabbage, parsley, chia seeds, beans, and buckwheat (Banwo et al., 2021).

Flavan-3-ols (Catechins) are found primarily in green tea, with powerful antioxidant and neuroprotective effects and an ability to modulate central nervous system pathways (Andrade and Assunção, 2015; Al-Khayri et al., 2022). Aside from tea (especially green and black tea), they can be found also in cocoa and its products, grapes, red wine, apples, and strawberries (Banwo et al., 2021).

Anthocyanidins are plant pigments with health-promoting properties, and often found in berries, grapes, apples, and purple vegetables. They have an oxygenated heterocyclic C ring and aromatic A and B ring structure, and cyanidin, delphinidin, pelargonidin, and malvidin are among their common forms (Burton-Freeman et al., 2016; Khoo et al., 2017). Proanthocyanidins are present in grape skin, red wine, berries, chocolate, and *Ginkgo biloba*, and have antioxidant and chronic disease-preventing roles that can be attributed to their polymeric flavonoid structures (Kooshki et al., 2023). Red and black beans, strawberries, cherries, grapes, pecans, Peruvian purple maize, red cabbage, red wine, red onions, and sorghum are common food sources (Banwo et al., 2021).

Phytoestrogens include isoflavones, which are mostly found in soy products. Depending on the situation, genistein and daidzein can modify steroid receptors and show either estrogenic or antiestrogenic activity (Mukund et al., 2017; Křížová et al., 2019). The main sources of isoflavones include soybean, meal made from soy, and other legumes (Banwo et al., 2021).

## 4. Therapeutic potential of flavonoids and phenolic compounds in neurodegenerative diseases

### 4.1. Alzheimer’s disease (AD)

Alzheimer’s disease (AD) is the most prevalent of the neurodegenerative diseases and has a multifactorial pathogenesis (Feigin et al., 2020). According to the World Health Organization (WHO), approximately 50 million people around the world have dementia, 60–80% of whom also have AD, and these figures are expected to rise exponentially (Nichols et al., 2019).

The etiology of AD involves the cumulative effects of modifiable and nonmodifiable risk factors. Among these, nonmodifiable risks include age, genetics, and family history, while modifiable risks include lifestyle, nutritional factors, physical inactivity, diabetes, hypertension, hypercholesterolemia, cerebrovascular disease, head trauma, and chronic stress. (Zhang et al., 2021b). The pathology of AD is characterized by the extracellular accumulation of amyloid-β (Aβ) peptides, which form senile plaques, and the intracellular aggregation of hyperphosphorylated tau proteins, which form neurofibrillary tangles (Hardy and Allsop, 1991). Other key mechanisms involved in the pathogenesis of AD include chronic neuroinflammation, mitochondrial dysfunction, oxidative stress, vascular dysfunction, impaired cerebral glucose metabolism, and cholinergic deficiency (Frost et al., 2017; Kinney et al., 2018; Butterfield and Halliwell, 2019). For example, microglia and astrocytes become activated in response to Aβ plaques and tau pathology, and trigger a persistent inflammatory response through intracellular activation of the NLRP3 (nucleotide-binding oligomerization domain-like receptor pyrin domain-containing 3) inflammasome (Van Zeller et al., 2021). All the above processes lead to neuron loss and the progression of the disease accelerates. Amyloid plaque accumulation caused by neuroinflammation has been recorded to begin 10–20 years before the onset of clinical symptoms (Bronzuoli et al., 2016; Le Page et al., 2018). Mild cognitive impairment is commonly regarded as the prodromal stage of AD and typically indicates early disease progression. Memory, attention, executive functioning, and visual-spatial abilities all deteriorate noticeably at this time. This process leads ultimately to dementia, which significantly lowers independence and quality of life (Potì et al., 2019).

To prevent disease onset, appropriate lifestyle interventions should be made in those with cognitive impairment before the clinical symptoms emerge. There are currently only two approved pharmacological treatment options for the treatment of AD: cholinesterase inhibitors and N-methyl-D-aspartate (NMDA) (Breijyeh and Karaman, 2020), although these medications provide only symptomatic relief rather than halting or reversing disease progression. Consequently, research efforts are increasingly focused on identifying the underlying mechanisms of AD, such as tau protein abnormalities, β-amyloid accumulation, inflammation, acetylcholinesterase activation (cholinergic dysfunction), and oxidative stress, and the development of effective therapies (Breijyeh and Karaman, 2020; Meng-zhen et al., 2022).

Recent studies have demonstrated that flavonoids may exert effects on several molecular targets involved in AD pathogenesis (Table 1) (Akinrinde and Adebiyi, 2019; Li et al., 2019; Wei et al., 2019; Dourado et al., 2020; Zhang et al., 2020; Nan et al., 2021; Singh and Garabadu, 2021; Kou et al., 2022; Tana and Nakagawa, 2022; Wang et al., 2024; Özdemir et al., 2025; Zhang et al., 2025a; Zhang et al., 2025b). Flavonoids have the potential to slow down neurodegeneration due to their strong antioxidant, antiinflammatory, antiapoptotic, and neurotrophic properties. Flavonoids, for example, exert a neuroprotective effect against the oxidative damage caused by reactive oxygen species (ROS) by increasing the expression of antioxidant defense enzymes via the Nrf2/ARE pathway (H. Khan et al., 2019). Preclinical studies have shown that quercetin can increase antioxidant enzyme HO-1 activity via the Nrf2 signaling pathway (Singh and Garabadu, 2021), while daidzein has been found to increase glutathione (GSH), superoxide dismutase (SOD), and catalase (CAT), and myricetin has been found to decrease ROS levels, with a protective effect against oxidative neuron damage (Wang et al., 2014; Wang et al., 2024).

Insulin resistance contributes to AD pathogenesis through neuroinflammation, Aβ aggregation, and tau hyperphosphorylation (Wei et al., 2021) by increasing GSK-3β enzyme activity. This stimulates NF-κB and NLRP3, leading to the release of proinflammatory cytokines such as tumor necrosis factor-α (TNF-α) and interleukin-6 (IL-6). Tau hyperphosphorylation occurs, and the above processes ultimately lead to increased neuroinflammation and neuronal damage (Wei et al., 2021). Flavonoids like chrysin and myricetin inhibit GSK-3β (Wang et al., 2024; Zhang et al., 2025b). Myricetin has been shown to decrease lipid peroxidation, DNA damage, and oxidative stress markers such as malondialdehyde (MDA) and thiobarbituric acid reactive substances (TBARS) through the activation of extracellular signal-regulated kinase (ERK) and GSK-3β signaling pathways, promoting cell survival and mitigating neuronal damage by modulating the PI3K/Akt and MAPK pathways (Wang et al., 2024).

Among the flavonoids, quercetin is considered one of the most promising for the treatment of AD due to its inhibitory effects on tau protein phosphorylation and Aβ aggregation. A study applying a triple transgenic AD model revealed that quercetin enhanced synaptic plasticity by increasing BDNF levels, reduced oxidative stress via Nrf2, and prevented the formation of neurofibrillary tangles. In other studies, myricetin, kaempferol, morin, apigenin, quercetin, and morin have been shown to decrease pathological amyloid deposition by blocking the activity of beta-secretase 1 (BACE1)– an essential enzyme in the production of Aβ (Shimmyo et al., 2008; Zhao et al., 2013; Baptista et al., 2014; Sabogal-Guáqueta et al., 2015; Dourado et al., 2020; Singh and Garabadu, 2021).

Proinflammatory cytokines like IL-1β cause neuroinflammation and neuronal damage by inhibiting such pathways as PI3K/Akt and MAPK/ERK, disrupting BDNF-mediated neuroprotection. Apigenin restores these pathways by increasing BDNF mRNA, suppressing IL-1β-induced neuroinflammation. (Venigalla et al., 2015; Dourado et al., 2020; Zhang et al., 2025a).

It has been suggested that soy isoflavones (such as genistein, daidzein, and equol) may have a neuroprotective effect on brain tissue due to their high affinity for the estrogen receptor beta (ERβ) (Gleason et al., 2015). Their estrogen-like effects and ability to enhance synaptic plasticity may offer protection against cognitive decline, particularly in postmenopausal women. In a clinical study, no progression of Aβ accumulation in the anterior cingulate gyrus was noted in patients receiving genistein supplementation, whereas the placebo group exhibited a significant increase (Table 2) (Viña et al., 2022). A positive correlation between plasma levels of equol and cognitive performance has been observed, and the effect is more pronounced when isoflavones are in their active, and more bioavailable, aglycone forms (Gleason et al., 2015).

Green tea catechins, including epigallocatechin gallate (EGCG), have demonstrated therapeutic potential for AD, with multiple mechanisms that include the disaggregation of Aβ aggregates, protection of mitochondria from oxidative damage, and modulation of the signaling pathways (Guo et al., 2017; Zhang et al., 2020; Nan et al., 2021). EGCG activates the cyclic AMP-response element binding protein CREB/BDNF/TrkB, PI3K/Akt/mTOR, and PI3K/Akt/eNOS pathways, thereby enhancing neuronal resistance to toxins such as 6-hydroxydopamine (6-OHDA) by regulating the genes involved in cell cycle and survival (Meng-zhen et al., 2022). This is particularly important for attenuating mitochondria-derived apoptotic signaling.

Resveratrol, a nonflavonoid polyphenol and phytoestrogen binding to estrogen receptors, protects neurons in AD by boosting cholinergic function, reducing β-amyloid toxicity, and preventing oxidative damage (Gu et al., 2021). It supports cellular defenses by regulating gene expression and energy metabolism through sirtuins, especially SIRT1, and inhibits tau hyperphosphorylation, BACE1 activity, neuroinflammation, and Aβ buildup via miRNA (Turner et al., 2015; Kou and Chen, 2017).

Resveratrol—particularly its trans-isomer and encapsulated form—has been shown in clinical studies to slow the decline of Aβ40 and Aβ42 levels in cerebrospinal fluid in patients with mild to moderate AD, reduce brain volume without functional loss (indicative of decreased Aβ accumulation), protect the blood-brain barrier by lowering (Matrix metalloproteinase-9) MMP9 levels, reduce neuroinflammatory markers (such as TREM2-related microglial activation and interleukins), decrease neuronal damage (e.g., Cathepsin D), and mitigate cognitive and functional decline (Turner et al., 2015; Moussa et al., 2017; Gu et al., 2021; Liu et al., 2025). Studies have shown that a 12-month course of resveratrol (500–2000 mg/day) may have potential therapeutic effects on the pathogenesis of AD (Table 2).

The bioavailability of flavonoids is a key factor determining their therapeutic potential. While apigenin, kaempferol, and quercetin exhibit moderate blood-brain barrier permeability, daidzein, genistein, and nobiletin demonstrate higher permeability (Shimazu et al., 2021). The low permeability of compounds such as rutin, fisetin, delphinidin, and cyanidin, however, may limit their neuroprotective effects (Rębas, 2025). Nevertheless, some polymeric flavonoids—such as proanthocyanidins—have been shown to accumulate as active metabolites in the brain with long-term use, thereby reducing AD pathology and supporting the processes related to learning and memory (Shimazu et al., 2021)

In conclusion, flavonoids exhibit multifaceted biological activities against AD and may offer clinically meaningful benefits. The few clinical studies of this subject to date have focused primarily on soy isoflavones and the nonflavonoid polyphenol resveratrol, and their generalizability has been limited by small sample sizes and their focus on patients with mild to moderate AD. Furthermore, two studies conducted with soy isoflavones have produced conflicting results, with one reporting that 100 g/day of soy isoflavones for 6 months had no effect on cognitive function, while another claimed that 120 g/day of genistein for 12 months improved cognitive function. These conflicting findings may be due to differences in the intervention period and dose, variations in the isoflavone type, and differences in the AD status of the patients. The results of preclinical studies indicate that the flavonoids quercetin, myricetin, chrysin, luteolin, apigenin, EGCG, and daidzein may slow the progression of the disease by inducing or reducing various mechanisms involved in AD pathogenesis. Their roles in enhancing antioxidant defenses, suppressing neuroinflammation, preserving synaptic function, and preventing the accumulation of Aβ and tau proteins highlight their potential as preventive and adjunctive therapeutic agents. Considering that interventions in the early stages of AD—such as at the onset of mild cognitive impairment—may delay the progression to dementia, flavonoid-based strategies represent a promising avenue for the management of AD. However, before definitive recommendations can be made, clinical trials of flavonoids that have shown promising effects in preclinical research should be conducted, and further investigations should be carried out.

### 4.2. Parkinson’s disease (PD)

Parkinson’s disease is the second-most common neurodegenerative disorder, and primarily affects older adults (Hou et al., 2019). According to the Global Burden of Disease Study 2015, PD is the fastest-growing neurodegenerative disorder (Feigin et al., 2017). PD is characterized by the pathological accumulation of the α-synuclein protein, neuroinflammation, oxidative stress, mitochondrial dysfunction, and reduced axonal transport in the substantia nigra, which is the center of dopamine production in the brain (Kalia and Lang, 2015). It contributes to the progressive degeneration of dopaminergic neurons and, consequently, to the loss of motor function (Man et al., 2021). These losses of motor function clinically manifest in PD patients as bradykinesia, resting tremor, rigidity, and postural instability (Krishnamoorthy et al., 2019). The currently approved pharmacological agents for the treatment of PD are levodopa, dopamine receptor agonists, and MAO-B inhibitors. However, such treatments can overstimulate the dopaminergic, serotonergic, and cholinergic pathways, with such potential adverse effects as nausea, and impulsive-compulsive behaviors. Moreover, these treatments provide only symptomatic relief; they do not halt the progression of the disease (Perdigão et al., 2023). These limitations of existing treatments have led researchers to look into ethnomedical and natural products as potential sources of neuroprotective agents that could modulate PD pathogenesis (Petramfar et al., 2020). In this context, flavonoids have emerged among the most promising compounds. According to one epidemiological study, a high flavonoid intake (especially flavonols, anthocyanins, and flavan-3-ols) and consumption of fruit and red wine are significantly associated with a reduced risk of mortality. Study participants who consumed three or more servings of fruit (especially berries) and red wine per week exhibited a lower mortality risk, suggesting that a flavonoid-rich diet may have beneficial effects on the prognosis of PD (Zhang et al., 2022).

Flavonoids have attracted attention as natural compounds due to their potential to target oxidative stress, mitochondrial dysfunction, neuroinflammation, and α-synuclein accumulation, as key mechanisms of PD pathology (Table 3). Notably, quercetin, myricetin, apigenin, EGCG, daidzein, naringenin, and hesperidin can contribute significantly to the prevention of oxidative stress-induced dopaminergic neuron loss (Levites et al., 2002; Antunes et al., 2014; B. Guo et al., 2016; Anusha et al., 2017; Siddique and Jyoti, 2017; Goes et al., 2018; Huang et al., 2018; Li et al., 2019; Sugumar et al., 2019; Tseng et al., 2020; Wang et al., 2022a; Gu et al., 2024; Liu et al., 2024; de Oliveira Vian et al., 2024; Yarim et al., 2025). These compounds reduce ROS formation and increase the activity of endogenous antioxidant enzymes such as GSH, SOD, CAT, and GPx through the activation of the Nrf2/ARE signaling pathway and its coactivator PGC-1α (whose expression is suppressed in PD) (González-May et al., 2024). Furthermore, myricetin, quercetin, and daidzein have been found to inhibit oxidative stress-induced dopaminergic neuronal damage via this pathway (Huang et al., 2018; Wang et al., 2022a; de Oliveira Vian et al., 2024).

Flavonoids play a critical role in suppressing neuroinflammation in addition to their antioxidant properties. Apigenin, myricetin, quercetin, luteolin, and naringin, for example, can effectively inhibit the production of proinflammatory cytokines (TNF-α, IL-1β, IL-6) in lipopolysaccharide (LPS)-activated microglial cells (Levites et al., 2002; Liu et al., 2008; Antunes et al., 2014; Zhang et al., 2015; Siddique and Jyoti, 2017; Goes et al., 2018; Huang et al., 2018; Sugumar et al., 2019; Gu et al., 2024; de Oliveira Vian et al., 2024; Yarim et al., 2025) by downregulating inflammation-related factors such as NF-κB and inducible nitric oxide synthase (iNOS). They also reduce secondary neuronal damage and protect dopaminergic circuits by attenuating microglial hyperactivation, and luteolin in particular has been shown to support the survival of dopaminergic neurons in mesencephalic neuron-glia cultures through such antiinflammatory actions (Chen et al., 2008; Zhu et al., 2011).

Flavonoids confer protection against Parkinson’s disease (PD) through the inhibition of apoptosis in dopaminergic neurons, which is induced by oxidative stress and mitochondrial dysfunction. Compounds such as chrysin, EGCG, and apigenin have been shown in preclinical investigations to reduce neuronal death by downregulating proapoptotic proteins such as Bax and caspase-3, while concurrently maintaining antiapoptotic proteins, including Bcl-2, Bcl-w, and Bcl-xL (Zhang et al., 2015; Guo et al., 2016; Anusha et al., 2017; Goes et al., 2018; Li et al., 2019; Tseng et al., 2020; Liu et al., 2024; Yarim et al., 2025). Among these, chrysin has also been found to support neuron survival by activating myocyte-specific enhancer factor 2D (MEF2D) (B. Guo et al., 2016).

Mitochondrial dysfunction leads to PD progression and neuron loss by increasing ROS levels, ATP depletion, and the activation of proapoptotic caspase-3 (Moon and Paek, 2015). Flavonoids show neuroprotective effects by improving mitochondrial dysfunction. Preclinical studies have shown that resveratrol, myricetin, and EGCG can improve ATP synthesis, promote mitochondrial biogenesis, and stabilize vital cellular processes (Levites et al., 2002; Zhao et al., 2017; Huang et al., 2018; Tseng et al., 2020; Gu et al., 2024).

Misfolded α-synuclein aggregation is the most characteristic feature of PD, and flavonoids such as myricetin, quercetin, and EGCG have shown significant promise in preventing α-synuclein fibrillation and promoting the disintegration of preexisting fibrils (Guo et al., 2017; Huang et al., 2018; Tseng et al., 2020; Gu et al., 2024; de Oliveira Vian et al., 2024). These striking impacts on the pathogenic aggregation mechanisms hold great promise for the creation of novel PD treatments; however, pharmacological constraints such as low stability, poor absorption, and limited oral bioavailability limit the therapeutic efficiency of flavonoids like EGCG for the treatment of PD. Encouragingly, several strategies like nanoparticle-based delivery and structural modifications have been developed recently to overcome these restrictions (Wang et al., 2022b).

Preserving the structural and functional integrity of dopaminergic neurons is another significant characteristic of flavonoids. Flavonoids such as myricetin, quercetin, apigenin, and genistein can enhance dopamine synthesis and release by boosting the expression of dopaminergic receptors such as tyrosine hydroxylase (TH), the dopamine transporter (DAT), and the dopamine D2 receptor (D2R), (Liu et al., 2008; Zarmouh et al., 2016; Anusha et al., 2017; Goes et al., 2018; Huang et al., 2018; Li et al., 2019; Gu et al., 2024; Liu et al., 2024; Yarim et al., 2025). The viability of dopaminergic neurons is based on such neurotrophic factors as BDNF and GDNF, which can be elevated by apigenin and chrysin (Anusha et al., 2017; Goes et al., 2018; Li et al., 2019; Liu et al., 2024; Yarim et al., 2025).

In conclusion, preclinical studies have reported flavonoids (quercetin, myricetin, chrysin, luteolin, apigenin, EGCG, naringenin, hesperidin, isoflavones, daidzein, genistein) to be promising as neuroprotective agents in PD due to their ability to target multiple pathological processes. Their antioxidant, antiinflammatory, mitochondria-preserving, antiapoptotic, and protein-aggregating properties collectively contribute to neuronal survival. The multidimensional nature of these compounds highlights their therapeutic potential, both as adjuncts to current pharmacological treatments, and as alternative disease-modifying strategies. Since all studies of this subject are at the preclinical level, however, further clinical studies are needed before flavonoid-based treatments can be recommended for PD patients.

### 4.3. Huntington’s disease (HD)

Huntington’s disease (HD) is a genetic neurodegenerative disorder found in all human populations, though its prevalence varies significantly among ethnic groups. People of Caucasian descent show relatively high prevalence rates, ranging from 10.6 to 13.7 cases per 100,000 people (Evans et al., 2013). HD is characterized by progressive motor disorders, cognitive decline, and psychiatric problems. Although the central nervous system is the most significantly affected, peripheral systems, particularly skeletal muscle, are also involved. The condition most commonly manifests as mitochondrial dysfunction in skeletal muscle, fibroblasts, and lymphoblasts (Neueder and Orth, 2020).

The Huntingtin (HTT) protein is expressed in nearly all cell types, and its mutant form is believed to play a central role in both the neurological and systemic manifestations of HD. The condition arises from an abnormal expansion of CAG trinucleotide repeats in exon 1 of the *HTT* gene, located on chromosome 4p16.3, leading to the production of a misfolded, toxic form of the Huntingtin protein (~348 kDa), which is widely expressed across various cell types. The mutant Huntingtin (mHTT) protein exerts harmful effects on both the nervous system and peripheral tissues, disrupting cellular homeostasis, accelerating mitochondrial aging, and impairing mitochondrial quality control systems, particularly during the early stages of the disease (Aldous et al., 2024).

Neuroprotective benefits of natural bioactive substances have been reported, focusing on different cellular processes that contribute to the pathophysiology of HD, including oxidative stress, excitotoxicity, mitochondrial dysfunction, and neuroinflammation. These substances may also lessen neuronal injury by modifying such proteostasis processes as autophagy and lysosomal breakdown (Gadade et al., 2024). The multitarget effects of natural bioactives when applied for the treatment of HD, particularly in a preclinical research setting, are presented in Table 4.

A recent study reported on the therapeutic potential of several phenolic and polyphenolic substances, with particular focus on HD pathogenesis-related processes, including neuroinflammation, oxidative stress, mitochondrial dysfunction, and HTT protein aggregation (El-Abhar et al., 2018; Elbaz et al., 2025). Among the compounds examined, morin demonstrated significant neuroprotective effects by suppressing necroptosis (RIPK1/3-MLKL), apoptosis (caspase-3), and neuroinflammation (TNF-α, GFAP) in rat models induced by 3-NP (El-Abhar et al., 2018; Elbaz et al., 2025). Morin was also noted to modulate such HD-related processes as mitophagy, ER stress, and mitochondrial dysfunction by inhibiting the mTOR/IRE1α/JNK and IP3R-VDAC pathways (El-Abhar et al., 2018). In a further study, the glycosylated form of chrysin (LQFM280) enhanced mitochondrial stability, balanced SDH and cholinesterase activity, and reduced oxidative damage in both cell culture and mouse models (Pereira et al., 2025).

Luteolin suppressed mHTT aggregation and reduced oxidative stress levels in both neuroblastoma cells (Ramadan et al., 2023; El-Emam et al., 2024) and a *Drosophila* model (Siddique et al., 2021). Rutin, on the other hand, exhibited neuroprotective effects in a *C. elegans* model through its antioxidant and metal-chelating activities against metal (Cu/Zn)-induced toxicity, and was noted to reduce polyglutamine aggregates (Cordeiro et al., 2023). Other flavonoids such as fustin and butin also enhanced endogenous antioxidant defense, including GSH, SOD, and CAT, and exhibited beneficial effects on inflammation and mitochondrial functions by reducing TNF-α, IL-1β, and lipid peroxidation products in HD models (Alshehri et al., 2022; Bin-Jumah et al., 2022). When encapsulated in PEG-PLGA nanoparticles, EGCG demonstrated improved bioavailability and resulted in significant improvements in motor deficits and depressive behaviors (Cano et al., 2021). Naringenin contributed to maintaining neurochemical balance by regulating MAO and serotonin levels and suppressed astrocytic activity by reducing GFAP expression (Salman et al., 2022).

7,8-Dihydroxyflavone (7,8-DHF) is a TrkB agonist that may play a significant role in neurogenesis, synaptic plasticity, and cell survival through its BDNF-mimetic effect (Paul et al., 2021). Similarly, compounds such as morin and chrysin can support synaptic integrity and neuronal function by activating the BDNF/TrkB/AKT/CREB pathway (El-Abhar et al., 2018; Mohamed et al., 2023). Bains et al. (2022) reported that ellagic and vanillic acids suppressed such pathological processes as oxidative stress, neuroinflammation, and mitochondrial dysfunction in an in vivo HD model, and reduced neuronal damage via AChE and caspase-3 inhibition. On the other hand, Pérez-Arancibia and colleagues reported that *Ugni molinae* extract reduced Huntingtin protein aggregation and modulated the expression of autophagy-related proteins in an in vitro cell model (Pérez-Arancibia et al., 2021).

It has been shown that flavonoids, as well as compounds such as astaxanthin, berberine, curcumin, and sulforaphane, enhance antioxidant systems by activating the Nrf2 pathway, reduce inflammation by inhibiting NF-κB, and support proteostasis via heat shock proteins (HSPs) (Silakari et al., 2024). Several studies to date have reported that these compounds exert neuroprotective effects by modulating key cellular signaling pathways – particularly PI3K/Akt/FoxO-1, JAK/STAT, IL-6/STAT3, and GSK-3β/CREB – stabilizing mitochondrial functions, enhancing ATP production, and suppressing neuronal death (Cianciulli et al., 2020; Li et al., 2021; Shawki et al., 2021; Rasheed and Ibrahim, 2022; Kooshki et al., 2023). In preclinical studies, flavonoids such as hesperidin, kaempferol, luteolin, and EGCG have been shown to significantly improve motor dysfunction, cognitive deficits, and oxidative stress parameters in HD models induced by 3-nitropropionic acid (3-NP) (Menze et al., 2012; Kumar et al., 2021; Islam et al., 2024). In particular, hesperidin reduced MDA levels, regulated antioxidant enzymes such as SOD and CAT, and limited nitric oxide production. Kaempferol inhibited NF-κB expression, thereby reducing the release of cytokines such as IL-1α, TNF-α, and C1q, consequently preventing the formation of A1 astrocytes, and also exhibited antiinflammatory effects by promoting microglial polarization toward the M2 phenotype (Darwish et al., 2023). These findings indicate that phenolic natural compounds, especially flavonoids, possess significant potential in alleviating HD symptoms and modulating underlying neuropathological processes. Although these results are promising, it should be kept in mind that they are based on preclinical studies with limited evidence.

In HD, symptoms such as dysphagia, weight loss, and gastrointestinal dysfunction can severely impact nutritional status, and while tube feeding offers some limited benefits, it comes with additional health risks. Human studies have shown the efficacy of dietary and supplement-based therapies to be limited, despite their encouraging outcomes in animal models. Thus, to better understand metabolic processes and direct nutritional treatments in HD, omics-based research is required (Gaba, 2025). Randomized controlled studies (RCTs) of such dietary supplements as vitamin E (d-alpha-tocopherol), omega-3 fatty acids (ethyl-eicosapentaenoic acid), coenzyme Q10, and creatine have yet to produce encouraging findings in terms of their effectiveness (Peyser et al., 1995; Huntington Study Group TREND-HD Investigators 2008; Ferreira et al., 2015; Hersch et al., 2017).

### 4.4. Amyotrophic lateral sclerosis (ALS)

Amyotrophic lateral sclerosis (ALS) is a progressive and lethal neurodegenerative illness that is characterized by the selective death of motor neurons in the brain, brainstem, and spinal cord, with hallmarks of axonal loss in the lateral spinal cord column (lateral sclerosis) and muscle loss (amyotrophy). Patients suffer from growing muscle weakness and paralysis as motor neuron loss worsens, and respiratory failure usually leads to death 3–5 years after the onset of symptoms (Chiò et al., 2009).

Although motor neurons are the primary target of ALS, other neuronal types and glial cells are also affected, contributing to extensive neurodegeneration. This cellular destruction leads to severe muscular atrophy, loss of motor function, and eventually death – most frequently from respiratory complications. The exact origins of ALS are still unknown, despite much research, and there is as yet no cure or effective treatment for the disease (Zhou and Xu, 2023).

The accumulation of misfolded proteins, genetic mutations, mitochondrial dysfunction, impaired autophagy, chronic neuroinflammation, altered RNA metabolism, disrupted axonal transport, and alterations in the gut microbiome are some of the pathological mechanisms thought to play a role in the development of ALS (Nguyen et al., 2018; Mitra et al., 2019; Zhou and Xu, 2023). Chromosome 9 open reading frame 72 (*C9orf72*), TAR DNA-binding protein (*TARDBP*), superoxide dismutase 1 (*SOD1*), and fused in sarcoma (*FUS*) are essential genes frequently linked to the pathophysiology of ALS, all of which are involved in the dysregulation of RNA metabolism. In addition to disrupting RNA processing, mutations in *C9orf72, TARDBP*, and *SOD1* also affect protein homeostasis. Specifically, mitochondrial malfunction and elevated oxidative stress are closely associated with mutant SOD1 (Nguyen et al., 2018), and mutant forms also play an role in disease pathogenesis by promoting protein aggregation, mitochondrial dysfunction, and motor neuron loss.

Numerous flavonoids have been shown in molecular investigations (Table 2) to bind to the mutant SOD1 protein and prevent pathological aggregation. The D101G mutant SOD1 protein has been investigated in molecular dynamics simulations, revealing that hesperidin and epigallocatechin gallate (EGCG) bind to the mutant, decrease β-sheet formation, stabilize hydrogen bonds, and improve the protein’s structural integrity (Maitham Qassim et al., 2023). Analyses of the A4V mutant version of SOD1 have also revealed the ability of the flavonoid morin to enhance structural stability, inhibit amyloidogenic activity, and halt aggregation (Srinivasan and Rajasekaran, 2018). Kaempferol, and its derivative kaempferide, have shown enhanced conformational flexibility, decreased β-strand content, and a high binding affinity, especially against the *SOD1G85R* mutant. These substances facilitate the clearance of misfolded proteins by promoting autophagy and activating the AMPK-mTOR pathway (Ueda et al., 2017; Srinivasan and Rajasekaran, 2018). Furthermore, in studies of N2a cell lines, kaempferol was found to prevent the formation of mitochondrial superoxide and dramatically decreased aggregation levels, based on which, kaempferol may be considered a viable natural treatment option for ALS.

The aggregation-inhibiting effects of flavonoids have been supported by in silico and in vivo studies (Table 5). Noorbakhsh et al. report (Noorbakhsh Varnosfaderani et al., 2025) that silybin and pelargonidin reduced β-sheet content, reorganized hydrogen bonds, and provided high structural stability in the free energy landscape of the E21K mutant SOD1. In a similar vein, an in vivo study by Koza (2022) using the *hSOD1G93A* transgenic mouse model showed that treatment with protocatechuic acid (PCA) suppressed gliosis, reduced motor neuron degeneration, and preserved neuromuscular junction integrity. According to Koza (2022), PCA administration (100 mg/kg) extended the median survival of hSOD1G93A mice from 121 to 133 days, corresponding to an approximate 12-day increase compared with untreated controls.

The therapeutic potential of natural polyphenols against ALS has been the subject of intensive study in recent years, revealing that phenolic compounds exert protective effects in ALS pathophysiology through mechanisms such as the activation of antioxidant defense systems, inhibition of protein aggregation, suppression of neuroinflammation, prevention of mitochondrial dysfunction, and preservation of synaptic plasticity. Baicalein and rutin have been noted to reduce mutant SOD1 protein aggregation, protect motor neurons, and improve motor functions (Du et al., 2024; Ting et al., 2025). EGCG directly interacted with TDP-43 and mutant SOD1, inhibiting early-stage protein aggregation and contributing to the restoration of mitochondrial functions

(Kumar et al., 2021; Morando et al., 2025). Apigenin exhibited cytoprotective effects through ALDH1A2 and activated the Nrf2/ARE signaling pathway to inhibit oxidative stress and apoptosis (Liang et al., 2024); and icariin significantly improved neurotoxic injuries that resembled those observed in previous studies by activating the SIRT1/Nrf2/HO-1 axis (Sharma et al., 2024). Gallic acid and morin have been identified as effective agents preventing misfolded protein-related pathologies by blocking the formation of SOD1 filaments (Srinivasan and Rajasekaran, 2018; Baek et al., 2023). Additionally, liquiritin apioside stabilized apo-SOD1 dimers, slowed conformational destabilization, and prevented aggregation (Zhao et al., 2022).

Nutritional status and lifestyle factors have also been reported to influence the progression of ALS. Natural antioxidants such as EGCG, coenzyme Q10, melatonin, alpha-lipoic acid, L-carnitine, and *Ginkgo biloba*, along with low-to-moderate intensity exercise, may offer potential benefits in slowing disease progression. In contrast, calorie restriction, malnutrition, and high-intensity exercise may lead to adverse outcomes (Patel and Hamadeh, 2009). These findings point to the potential benefits of flavonoid-based bioactive molecules for the treatment of ALS through multitarget strategies, although advanced in vivo and human studies are required to validate the clinical relevance of these preclinical findings.

## 5. Challenges and limitations

### 5.1. Low bioavailability and metabolic transformation

The health benefits of phenolic compounds can be attributed primarily to their biotransformation-derived metabolites. The necessary bioavailability for their effectiveness is influenced by various factors, including dietary composition, chemical structure, solubility, molecular size, release from the food matrix, and post-digestive and absorption metabolic processes (Czubinski et al., 2019; Chen et al., 2022).

Absorption of phenolic compounds in the small intestine is generally low, with only approximately 5–10% of ingested polyphenols being absorbed, depending on their chemical structure. The primary factors contributing to the low bioavailability of flavonoids include poor solubility, limited intestinal permeability, the rapid and complex nature of enzymatic metabolism, and their interactions with the gut microbiota (Leonarduzzi et al., 2010; Teng and Chen, 2019; Chen et al., 2022). Flavonoids are typically ingested in glycoside form and are absorbed in the intestine after being converted into their aglycone form. This conversion depends on the β-glucosidase activity of intestinal microbiota and represents a significant barrier to absorption. Following absorption, flavonoids undergo phase II metabolic reactions in the liver – such as glucuronidation, sulfation, and methylation – which limit the systemic circulation of their active forms (Chen et al., 2022). Flavonoid bioavailability is also significantly influenced by the composition of the foods in which they are consumed. For example, dietary fiber, particularly its soluble forms, can prolong the gastric emptying time and physically entrap flavonoids, thereby reducing their absorption (Pérez-Jiménez et al., 2013). In contrast, dietary lipids can enhance the absorption of apolar phenolic compounds by promoting their micellization. For example, it has been shown that the bioaccessibility of quercetin increases when consumed with fat-containing meals (Guo et al., 2013). In the case of such compounds as resveratrol, this effect has yielded conflicting results (la Porte et al., 2010; Guo et al., 2013).

The bioavailability of flavonoids varies significantly depending on their subclasses and structural characteristics. Isoflavones (such as genistein and daidzein), flavanones, catechins, and quercetin glucosides generally exhibit higher absorption, whereas condensed tannins, anthocyanins, and tea catechins are characterized by notably low bioavailability (Eghbaliferiz and Iranshahi, 2016; Naeem et al., 2022). Structurally larger and more polymeric compounds, as well as esterified or highly glycosylated forms, are generally more difficult to absorb. Even at high oral intake levels, the expected pharmacological effects of flavonoids are often not achieved in plasma or tissue concentrations, which can be attributed primarily to poor absorption, metabolic transformation, interindividual biological variability, and rapid elimination. Additionally, the inability of flavonoids to exhibit tissue-specific distribution further limits their therapeutic efficacy (Maan et al., 2020). Considering all the above factors, achieving adequate bioavailability of flavonoids through conventional oral administration can be highly challenging, representing a significant obstacle to the clinical application of flavonoids, particularly for neuroprotective or other therapeutic purposes.

### 5.2. Inter-individual variability in absorption and response

There are many substantial inter-individual variations in the biological responses to flavonoids (Albuquerque et al., 2021; Eseberri et al., 2022; Faysal et al., 2024). These include the physicochemical characteristics of the flavonoids (such as their solubility), dosage, form, gastrointestinal transit time, gastrointestinal pH, blood flow rate, enzyme activity, and transporter protein levels (Zhao et al., 2019; Naeem et al., 2022). Solute carrier (SLC) and ATP-binding cassette (ABC) transporters are essential components of the transporters affecting flavonoid bioavailability, limiting the oral bioavailability of flavonoids by controlling their permeability, stability, and solubility. Furthermore, metabolic enzymes, including those belonging to the cytochrome P450 (CYP450) family, are responsible for inter-individual metabolic rate differences (Oostendorp et al., 2009).

The gastrointestinal microbiota plays a central role in flavonoid metabolism. Specifically, intestinal bacteria participate in the critical preabsorptive step, converting flavonoid glycosides into aglycones. When the unabsorbed part of flavonoids reaches the colon, they undergo such microbial enzymatic changes as ring fission, deglycosylation, demethylation, and dihydroxylation, which produce more minor, bioactive metabolites (Van Duynhoven et al., 2011; Wan et al., 2021). Approximately 20% of the polyphenols that are consumed orally are thought to be absorbed in the small intestine, while the majority reach the colon. Phenolic chemicals may exert their effects through fundamental mechanisms that involve prolonged exposure and interaction with the intestinal flora. These changes may directly impact the systemic bioavailability and therapeutic effectiveness of flavonoids. For instance, the gut microbiota-mediated transformation of soy-derived isoflavones into more potent derivatives underscores the importance of individual microbial composition (Eseberri et al., 2022; Naeem et al., 2022). The gut microbiota is formed under the influence of many different factors such as genetics, age, sex, ethnicity, physical activity, medication use, and nutrition (Eseberri et al., 2022).

### 5.3. Standardization and dosing issues

In order to maximize the health benefits of phenolic compounds, it is essential to accurately determine the dosage, dosing frequency, and timing of administration, as well as appropriate compounds (Zhao et al., 2019; Faysal et al., 2024). The traditional pharmacological paradigm of “higher dose equals greater effect” is not applicable for many phenolic substances. According to the hormetic response model, smaller doses may have positive effects, while larger amounts may be ineffective or even hazardous (Eseberri et al., 2022). For example, it has been found that resveratrol has an antiobesity impact at 30 mg/kg; however, the effect is decreased at 60 mg/kg (Macarulla et al., 2009). In the same way, genistein, kaempferol, and curcumin have shown pro-oxidant effects at greater concentrations while exhibiting antioxidant activity at lower amounts (Cao et al., 2006; Oh et al., 2006; El Touny and Banerjee, 2009). There are substantial variations in the nature and levels of active compounds stemming from the plant origin of flavonoids, which makes standardizing doses quite challenging. The flavonoid content of plants varies based on several factors, such as plant species, growing conditions, extraction method, and storage, while variations in glycosylation, molecular structure, and formulations directly affect absorption and bioavailability (Albuquerque et al., 2021; Chen et al., 2022). These structural and formulation-related factors raise questions about the consistency of the results of clinical trial and make the establishment of trustworthy dose-response correlations more challenging. A lack of information on safe and effective dose ranges still hampers the routine therapeutic use of phenolic compounds in clinical settings (Maan et al., 2020; Saleem et al., 2022).

The absorption and metabolism of phenolic compounds can be significantly impacted by several factors, including whether the dose is taken all at once or spread throughout the day, whether it is taken with or without food, and even the time of day it is given. Specifically, metabolic processes like glucuronidation are affected by diurnal fluctuations, underscoring the significance of timing (Eseberri et al., 2022).

Though the goal of developing new delivery systems is to increase the solubility, stability, and absorption potential of phenolic compounds, although studies are continuing to determine the best dosage schedules, develop customized administration procedures, and incorporate these compounds into clinical practice (Chen et al., 2022). To ensure clinical efficacy and safety, the development of comprehensive and standardized dosing methods that consider the bioavailability, metabolism, and individual response profiles of phenolic compounds is essential.

### 5.4. Regulatory and safety considerations

Flavonoids are considered dietary supplements and so are not subject to the strict regulatory approval required for pharmaceuticals, which can raise safety and toxicity concerns (Solnier et al., 2023). Regulatory approval for therapeutic use demands solid safety and efficacy data, yet few phenolic compounds have been granted approval such regulatory bodies as the FDA or EFSA (Zhao et al., 2019; Albuquerque et al., 2021). While generally considered safe, some flavonoids can be toxic in high doses (Hytti et al., 2017; Liu et al., 2019), with effects influenced by dosage, usage duration, and individual genetic or metabolic factors (Faysal et al., 2024). It is essential to thoroughly assess the immunological responses, estrogenic effects, and possible developmental toxicity of their use in children, adolescents, and multiple/chronic drug users (Zhang et al., 2021a; Barreca et al., 2023).

## 6. Future perspectives and research directions

Strategies that account for interindividual variability—such as genetic profiling and microbiota analysis—hold promise for optimizing the therapeutic efficacy of flavonoids. Such strategies, summarized in Table 6, can enhance the predictability of individual responses and support the personalization of treatment regimens (Kopalli et al., 2025). Future longitudinal studies are expected to provide more precise data on the long-term efficacy and safety of flavonoids, as well as their impact on cognitive function and quality of life. In the meantime, improving bioavailability, clarifying dose–response relationships, and developing targeted delivery systems remain priority areas for the achievement of clinical success (Teng and Chen, 2019)

## 7. Conclusions

In this comprehensive examination of the multifaceted neuroprotective effects of flavonoids and phenolic compounds in the context of neurodegenerative diseases, flavonoids have been shown to be effective in targeting the key pathological mechanisms common to neurodegenerative diseases. These mechanisms include oxidative stress, mitochondrial dysfunction, neuroinflammation, and protein aggregation. In particular, quercetin, myricetin, apigenin, luteolin, EGCG, genistein, and morin have demonstrated significant abilities to improve cognitive function, suppress inflammatory response, and inhibit neuronal cell death. However, there are several challenges to be overcome, such as low bioavailability, poor pharmacokinetics, and metabolic instability, which continue to limit the widespread clinical application of these natural compounds. It should be emphasized that most of the current evidence has been garnered through preclinical models, and the translation of these findings to humans remains limited due to differences in physiology, dosage, and metabolic processes.

To fully harness the therapeutic potential of flavonoids, several strategic recommendations are necessary. First, given that most existing data are derived from in vitro and animal studies, there is an urgent need for well-designed, large-scale clinical trials. Considering the evidence suggesting that flavonoids are most effective during the preclinical or early symptomatic stages of disease, early diagnosis combined with timely intervention may help delay disease progression. Furthermore, the use of advanced pharmaceutical technologies – such as nanoparticle-based delivery systems, liposomes, and structural modifications – should be prioritized to overcome bioavailability-related limitations. At a public health level, promoting the regular consumption of flavonoid-rich foods (e.g., fruit, vegetables, green tea, soy products) could contribute to a nutritional strategy targeting neurodegenerative diseases. Finally, combining flavonoids with conventional pharmacological therapies may not only enhance treatment efficacy, but also mitigate drug-related side effects. In this regard, a multidisciplinary and translational approach is essential if the full potential of flavonoids in the prevention and management of neurodegenerative diseases is to be realized.

## Figures and Tables

**Figure:**
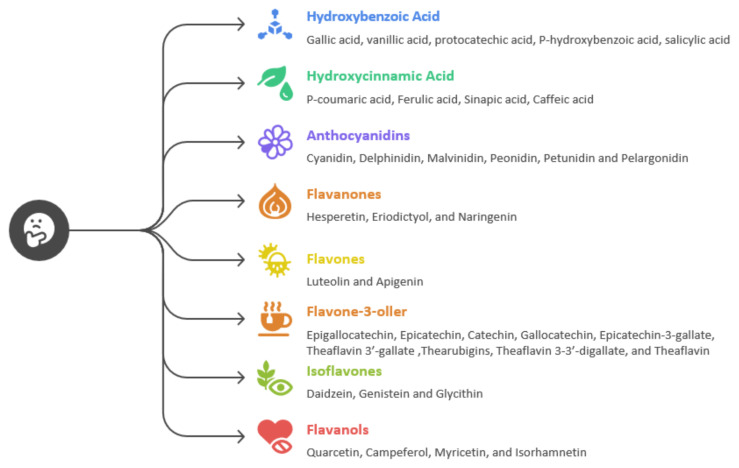
Classification of phenolic acids and polyphenols (adapted from Banwo et al., 2021)

**Table 1:** Preclinical evaluation of the AD-preventive effects of flavonoids.

Phenolic compounds		AD preventive mechanisms		Study designs	References
Flavonoids		Reduced	Induced		
**Flavonols**	Quercetin	β-amyloidosis Tauopathy Astrocyte overactivation (GFAP) Microgliosis BACE1-mediated cleavage of APP Learning and memory deficits Scattered senile plaques Mitochondrial dysfunction Reactive oxygen species	Improved performance in learning and spatial memory tasks AMP-activated protein kinase (AMPK) activity α7nAChR/Nrf2/HO-1-mediated neuroprotection	In vivo	(D. Wang et al., 2014; Sabogal-Guáqueta et al., 2015; Singh and Garabadu 2021)
	Myricetin	Aβ aggregation BACE1 activity Tau phosphorylation ROS generation Lipid peroxidation DNA oxidation Mitochondrial dysfunction via the associated GSK3β and ERK 2 signaling pathways.	Improved performance in cognition and learning	In vivo	(L. Wang et al., 2024)
**Flavones**	Chrysin	Lipopolysaccharide (LPS)-induced microglia activation NO, IL-6, and TNF-α Aβ aggregation Tau hyperphosphorylation βACE1 and GSK-3β		In vitro & In vivo	(Li et al., 2019; Zhang et al., 2025b)
	Luteolin	Astrocyte overactivation (GFAP) LPS-induce activity of NF-κB TNF-α, IL-1β, IL-6, NO, COX-2, iNOS, PGE2 Brain tissue ER stress markers (GRP78 and IRE1α) AChE activity		In vitro & In vivo	(Zhu et al., 2011; Akinrinde and Adebiyi 2019; Kou et al., 2022; Tana and Nakagawa 2022)
	Apigenin	Microglial activation Caspase-3 activation IL-6 BACE1 and β-CTF levels (Aβ burden prevention)	BDNF ERK/CREB/BDNF pathway Neurons and astrocytes integrity	In vitro & In vivo	(L. Zhao et al., 2013; Dourado et al., 2020)
**Flavanols**	EGCG	Escape latency Aβ42 level AChE activity Tau hyperphosphorylation APP, BACE1 and Aβ1–42 expression	BDNF Improved performance on spatial memory	In vivo	(Y. Guo et al., 2017; S. Zhang et al., 2020; Nan et al., 2021)
**Isoflavones**	Daidzein	MDA Aβ-induced oxidative stress Caspase-3	Improved performance on memory and learning SOD, CAT, and GSH	In vitro & In vivo	(Mao et al., 2007; Wei et al., 2019; Özdemir et al., 2025)

**Aβ:** Amyloid beta, **APP:** Amyloid precursor protein, **BACE1:** Beta-site APP cleaving enzyme 1, **β-CTF:** Beta-cleaved C-terminal fragment, **GFAP:** Glial fibrillary acidic protein , **GSK-3β:** Glycogen synthase kinase 3 beta, **ERK1/2:** Extracellular signal-regulated kinases 1 and 2, **CREB:** cAMP response element-binding protein, **AMPK:** AMP-activated protein kinase, **NF-κB:** Nuclear factor kappa-light-chain-enhancer of activated B cells, **HO-1:** Heme oxygenase-1, **Nrf2:** Nuclear factor erythroid 2-related factor 2, **IRE1α:** Inositol-requiring enzyme 1 alpha, **GRP78:** Glucose-regulated protein 78, **α7nAChR:** Alpha-7 nicotinic acetylcholine receptor, **ROS:** Reactive oxygen species, **MDA:** Malondialdehyde, **NO:** Nitric oxide, **SOD:** Superoxide dismutase, **CAT:** Catalase, **GSH:** Glutathione, **IL-6:** Interleukin-6, **IL-1β:** Interleukin-1 beta, **TNF-α:** Tumor necrosis factor-alpha, **iNOS:** Inducible nitric oxide synthase, **COX-2:** Cyclooxygenase-2, **PGE2:** Prostaglandin E2, **AChE:** Acetylcholinesterase, **BDNF:** Brain-derived neurotrophic factor, **LPS:** Lipopolysaccharide.

**Table 2:** Clinical evidence on the effects of phenolic compounds (flavonoid/nonflavonoid) in AD symptomatology and pathogenesis.

Intervention	Sample size	Participants	Duration	Outcomes	Reference
**Flavonoids**					
120mg/day genistein	Treatment: 13 Placebo: 11	Prodromal AD patients	12 months	Improved in 2 cognitive test scores; stable Aβ in anterior cingulate	(Viña et al., 2022)
100mg/day soy isoflavone	Treatment:32 Placebo: 33	AD patients	6 months	No statistically significant differences were identified between treatment groups or between sexes in relation to cognitive function test outcomes.	(Gleason et al., 2015b)
**Nonflavonoids**					
Resveratrol (encapsulated) 500–2000 mg per day, with a 500 mg dose increase every 13 weeks.	Treatment: 19 Placebo: 19	Mild-to-moderate AD	12 months	↓ CSF MMP9, ↑ MDC, IL-4, FGF-2, plasma MMP10, ↓ IL-12P40, IL-12 P70 attenuated cognitive and functional decline (MMSE, ADCS-ADL) attenuated decline in CSF Aβ42 levels	(Moussa et al., 2017)
Trans-resveratrol (500 mg orally once daily)	Treatment: 15 Placebo: 15	Mild to moderate AD	12 months	↓ CSF MMP9, attenuated functional decline (ADCS-ADL), attenuated decline in CSF Aβ40 ↑Brain volume loss	(J. Gu et al., 2021)
Resveratrol (encapsulated) 500–2000 mg per day, with a 500 mg dose increase every 13 weeks.	Treatment: 30 Placebo: 21	Mild-to-moderate AD	12 months	↓ TREM2, MMP-9, Cathepsin D; ↑ angiogenin ↓Neuron-specific enolase and hyperphosphorylated neurofilaments attenuated decline in CSF Aβ40 ↑ Brain volume loss	(X. Liu et al., 2025)
Trans-Resveratrol (encapsulated) 500–2000 mg per day, with a 500 mg dose increase every 13 weeks.	Treatment: 56 Placebo: 48	Mild-to-moderate AD	12 months	attenuated decline in CSF Aβ40 ↑ brain volume loss attenuated functional decline (ADCS-ADL)	(Turner et al., 2015)

**AD:** Alzheimer’s disease, **CSF:** Cerebrospinal fluid, **MMP:** Matrix metalloproteinase, **MDC:** Macrophage-derived chemokine, **IL-4:** interleukin-4, **FGF-2:** fibroblast growth factor-2, **Aβ:** Amyloid beta, **MMSE:** Mini-mental state examination, **ADCS-ADL:** Alzheimer’s disease cooperative study–activities of daily living.

**Table 3:** Preclinical evaluation of the PD-preventive effects of flavonoids.

Flavonoids	PD preventive mechanisms		Study designs	References
	Reduced	Induced		
**Flavonols**				
**Quercetin**	TBARS, MDA, LPO IL-1B, IL-6 α-synuclein protein expression	Nrf2 protein Sirtuins (SIRT1) CAT, SOD, GSH, GPx PI3K	In vivo	(de Oliveira Vian et al., 2024)
**Myricetin**	LPS-induced microglia activation LPS-induced TH neuron damage IL-1β, TNF-α, IL-6 MAPKs and NF-κB signaling α-synuclein accumulation ROS, MDA	Motor function TH protein expression GSH, GPx Nrf2 pathway	In vivo & in vitro	(Huang et al., 2018; S. Gu et al., 2024)
**Flavones**				
**Chrysin**	LPS-induced microglia activation NO, iNOS expression NF-κb activation IL-1β, TNF-α, IL-6 MDA, LPO Antiapoptotic protein Bcl-2 MAO-B	Nrf2/HO-1 Antioxidant enzymes expression (HO-1, SOD, and CAT) MEF2D activation. Akt/GSK-3β/MEF2D pathway BDNF, GDNF expression TH+ neurons	In vivo	(Z. Zhang et al. 2015; B. Guo et al., 2016; Goes et al., 2018; Z. Li et al., 2019)
**Luteolin**	LPS-induced microglia activation LPS-induced decrease in [3H] dopamine uptake iNOS, COX-2 activation IL-1β, NO, PGE2, TNF-α		In vitro	(H. Chen et al., 2008; Zhu et al., 2011)
**Apigenin**	NF-κb, iNOS activation IL-1β, IL-6, IL-10, TNF-α, TGF-β Dopaminergic neuronal loss α-synuclein accumulation AChE enzyme activity LPO MAO-B Caspase-3, and caspase-9 activity	TH protein expression dopamine D2 receptor (D2R) expression GSH, GPx, GR, and GST PI3K/Akt/NF-κb pathway (TNF-α, IL-1β, and IL-6 inhibition) BDNF, GDNF expression	In vivo & in vitro	(Anusha et al., 2017; Siddique and Jyoti, 2017; K. Liu et al., 2024; Yarim et al., 2025)
**Flavanols**				
**EGCG**	NO, LPO Nitrite, TBARS, TNF-α, IL1β, IL-6, and caspase-3 Bcl-2, Bcl-w, and Bcl-x(L) α-synuclein fibrillation	SDH ETC enzymes PKC, ERK1/2 activities	In vivo & in vitro	(Levites et al., 2002; J. Zhao et al., 2017; Tseng et al., 2020)
**Flavanon**				
**Naringenin**	LPO levels, inos expression, mRNA concentrations of proinflammatory cytokines TNF-α and IL-1β	GR and CAT Behavioral performance	In vivo	(Sugumar et al., 2019)
**Hesperidin**	ROS and glutathione reductase activity	GPx and CAT activity	In vivo	(Antunes et al., 2014)
**Isoflavones**				
**Daidzein**,	ROS levels, LPS-induced apoptosis	GSK3β/Nrf2/ARE pathway. Nrf2 expression	In vivo	(Wang et al., 2022a)
**Genistein**	TH-immunoreactive neuron toxicity MAO-B	TH, dopamine transporter and Bcl-2 mRNA expression	In vivo & in vitro	(L. Liu et al., 2008; Zarmouh et al., 2016)

**PD:** Parkinson’s disease, **TBARS**: Thiobarbituric acid reactive substances, **MDA:** Malondialdehyde, **LPO:** Lipid peroxidation, **Nrf2:** Nuclear factor erythroid 2–related factor 2, **CAT:** Catalase, **SOD:** Superoxide dismutase, **GSH:** Glutathione, **GPx:** Glutathione peroxidase, **PI3K:** Phosphoinositide 3-kinase, **LPS:** Lipopolysaccharide, **TH:** Tyrosine hydroxylase, **TNF-α:** Tumor necrosis factor-alpha, **MAPKs:** Mitogen-activated protein kinases, **NF-κB:** Nuclear factor kappa B, **ROS:** Reactive oxygen species, **GR:** Glutathione reductase, **GST:** Glutathione S-transferase, **NO:** Nitric oxide, **iNOS:** Inducible nitric oxide synthase, **MEF2D:** Myocyte enhancer factor 2D, **MAO-B:** Monoamine oxidase B, **HO-1:** Heme oxygenase-1, **Akt:** Protein kinase B, **GSK-3β:** Glycogen synthase kinase 3 beta, **BDNF:** Brain-derived neurotrophic factor, **GDNF:** Glial cell line-derived neurotrophic factor, **COX-2:** Cyclooxygenase-2, **PGE2:** Prostaglandin E2, **TGF-β:** Transforming growth factor beta, **SDH**: Succinate dehydrogenase, **ETC:** Electron transport chain, **PKC:** Protein kinase C, **ERK1/2:** Extracellular signal-regulated kinases 1 and 2, **ARE:** Antioxidant response element.

**Table 4:** Mechanisms of action of flavonoid and phenolic compounds tested in Huntington’s disease models.

Reference	Bioactive compound	Study type	Mechanism of action	Main results
(Elbaz et al., 2025)	Morin Hydrate	In vivo Rat: HD model induced by 3-NP	Neuroinflammation Apoptosis Necroptosis Mitochondrial function,	TNF-α ↓, GFAP ↓ Caspase-3 and 8 ↓ RIPK1/3-MLKL ↓ SDH ↑
(Pereira et al., 2025)	LQFM280 (Chrysin + β-D-glucose tetraacetate)	In vitro Cells: SH-SY5Y, STHdhQ111/Q111 (mutant), STHdhQ7/Q7 (wild-type) cell lines In vivo Mice: HD model induced by 3-NP	Antioxidant Mitochondrial function Enzyme inhibition	Oxidative stress ↓ SDH ↓ (modulation) AChE ↓(modulation)
(El-Abhar et al., 2018)	Morin Hydrate	In vivo Rat In silico Molecular docking-based analysis of binding interactions with mTOR, IRE1α, JNK, and IP3R targets	Neuroinflammation Apoptosis Mitophagy Mitochondrial function Neurotrophic factors	mTOR/IRE1α/JNK↓ cytochrome c/caspase 3↓ PINK1/Ubiquitin/Mfn2 ↓ Caspase 3↓ MERC dysfunction p-VDAC1 ↑ p-PGC-1α ↑
(Ramadan et al., 2023)	Luteolin	In vitro Neuroblastoma cell line (160Q and 20Q) transfected with mutant Huntingtin (mHTT)	Protein aggregation Apoptosis Antioxidant	mHTT aggregates ↓, caspase-3 ↓ Nrf2–HO1 pathway↑
(Cordeiro et al., 2023)	Rutin	In vivo Model organism: Transgenic Huntington’s disease model in Caenorhabditis elegans	Antioxidant Protein aggregation Enzyme chelation	PolyQ aggregates ↓, motor dysfunction↓, neutralization of metal toxicity
(Mohamed et al., 2023)	Morin Hydrate	In vivo Rat: HD model induced by 3-NP	Neuroinflammation Antioxidant Enzyme inhibition, Neurotrophic factors Synaptic plasticity	NF-κB ↓ NF-α, IL-1β ↓ Glutamate/Calpain↓ BDNF/TrkB/AKT/CREB ↑
(Bin-Jumah et al., 2022)	Fustin	In vivo Rat: HD model induced by 3-NP	Antioxidant Neuroinflammation Neurotrophic factors	GSH, SOD, CAT ↑; MDA, Nitrite↓ TNF-α, IL-1β, COX↓ BDNF ↑
(Bains et al., 2022)	Ellagic & Vanillic Acids	In vivo Rat: HD model induced by intrastriatal quinolinic acid injection	Antioxidant Neuroinflammation Mitochondrial function Enzyme inhibition	GSH↑, MDA, nitrite↓; TNF-α,IL-6,NF-κB↓; Mitochondrial complexes ↑ AChE, Caspase 3 ↓
(Alshehri et al., 2022)	Butin	In vivo Rat: HD model induced by 3-NP	Antioxidant, Mitochondrial function Enzyme inhibition	MDA, nitrite↓; GSH↑, CAT, SOD ↑; SDH activity and ATP↑, AChE ↓, LDH ↓
(Pérez-Arancibia et al., 2021)	Ugni molinae extract	In vitro HD cell model based on polyQ-EGFP protein exp. in HEK293 cell	Protein aggregation Antioxidant	PolyQ aggregates ↓ EGFP aggregates ↓
(Salman et al., 2022)	Naringenin	In vivo Rat: HD model induced by 3-NP	Antioxidant, Neuroinflammation Enzyme modulation	GFAP ↓ MAO, 5-HT ↑ Motor function ↑
(Siddique et al., 2021)	Luteolin	In vivo Model organism: Transgenic Drosophila model of HD	Antioxidant Protein aggregation	Oxidative stress ↓ mHTT interaction, Climbing ability ↑;
(Cano et al., 2021)	EGCG-PLGA NPs	In vivo Mouse: HD model induced by 3-NP	Antioxidant, Neuroinflammation,	Motor dysfunction ↓; neuron loss ↓

**HD** (Huntington’s Disease), **3-NP** (3-Nitropropionic Acid),**EGFP** (Enhanced Green Fluorescent Protein), **mHTT** (Mutant Huntingtin), **SH-SY5Y** (Human Neuroblastoma Cell Line), **STHdhQ111/Q111** (Striatal-derived mutant Huntingtin cell line), **STHdhQ7/Q7** (Striatal-derived wild-type cell line), **SDH** (Succinate Dehydrogenase), **AChE** (Acetylcholinesterase), **GFAP** (Glial Fibrillary Acidic Protein, **IKK** (IκB kinase), **RIPK1/3** (Receptor-interacting protein kinases 1 and 3), **MLKL** (Mixed Lineage Kinase Domain-like Protein), **GSH** (Glutathione), **SOD** (Superoxide Dismutase), **CAT** (Catalase), **MDA** (Malondialdehyde), **COX** (Cyclooxygenase), **LDH** (Lactate Dehydrogenase), **TrkB** (Tropomyosin receptor kinase B), **AKT** (Protein Kinase B), **CREB** (cAMP Response Element-Binding Protein), **5-HT** (5-Hydroxytryptamine / Serotonin), **PGC-1α** (Peroxisome proliferator-activated receptor gamma coactivator 1-alpha), **VDAC1** (Voltage-Dependent Anion Channel 1), **PINK1** (PTEN-Induced Kinase 1), **Mfn2** (Mitofusin-2), **NPs** (Nanoparticles), **PLGA** (Poly(lactic-co-glycolic acid))

**Table 5:** The relationship between ALS and bioactive compounds.

Reference	Bioactive compound	Method	Mechanism of action	Main findings
(Ting et al., 2025)	Baicalein	In vitro (motor neuron cell line transfected with mutant SOD1-G93A); In vivo (SOD1-G93A mouse model)	Protein aggregation	Baicalein reduced SOD1 aggregates, preserving motor neuron density and motor function.
(Morando et al., 2025)	Epigallocatechin-3-gallate (EGCG)	In vitro (NMR, fluorescence, modelling)	Protein aggregation	EGCG inhibited TDP-43 aggregation by binding to RNA recognition motifs (RRMs) and increased protein stability.
(Liang et al., 2024)	Apigenin (APG)	In vitro (NSC34 cells); In vivo (SOD1-G93A mouse model)	Antioxidant, Enzyme induction (Nrf2), Apoptosis modulation	APG activated the ALDH1A2–Nrf2 pathway, suppressed oxidative stress and apoptosis, and improved motor function.
(Du et al., 2024)	Rutin	In vivo (SOD1-G93A mouse model)	Protein aggregation, Neuroinflammation	Rutin reduced SOD1 aggregation and glial activation, protecting motor neurons.
(Sharma et al., 2024)	Icariin	In vivo (MeHg-induced neurotoxic ALS model); In silico (binding to SIRT1, Nrf2, HO-1, TDP-43)	Antioxidant, Neuroinflammation, Enzyme induction (SIRT1, Nrf2, HO-1)	Icariin activated the SIRT1–Nrf2–HO-1 pathway, reducing inflammation and neurotoxicity.
(Baek et al., 2023)	Gallic acid	In vitro (filament assay, ITC, MD simulation)	Protein aggregation	Gallic acid both inhibited and dissolved SOD1 filament formation.
(Srinivasan & Rajasekaran, 2018)	Morin	In silico (protein–ligand interaction, molecular dynamics simulations)	Protein aggregation	Morin effectively inhibited A4V SOD1 aggregation and was proposed as a potential therapeutic agent.
(B. Zhao et al. 2022)	Liquiritin apioside	In vitro (Native ESI-IM-MS, ESI-MS/MS, CIU, fluorescence spectroscopy)	Protein aggregation, Protein stabilization	Liquiritin apioside stabilized apo-SOD1 conformation and inhibited aggregation.
(Yadav et al., 2022)	Apigenin (APG)	In vivo (MeHg-induced neurotoxic ALS model)	Antioxidant, Neuroinflammation, Enzyme inhibition (JNK/p38MAPK)	APG suppressed neuroinflammation and oxidative damage, providing behavioral and molecular improvements.
(Martín-Cámara et al., 2022)	Fasudil–ferulic/caffeic acid derivative (1d)	In vitro (MTT, RT-PCR; SOD1-ALS and sALS patient-derived lymphoblast cell lines)	Antioxidant, Enzyme induction (Nrf2), Enzyme inhibition (ROCK2)	Compound 1d enhanced antioxidant response via ROCK2 inhibition and Nrf2 activation, showing therapeutic potential.
(Kumar et al., 2021)	EGCG, Isorhamnetin, Kaempferol	In silico (molecular docking and dynamics simulation)	Protein aggregation, Protein stabilization	These polyphenols stabilized the I113T mutant SOD1 structure and prevented aggregation.

**ALS** (Amyotrophic Lateral Sclerosis), **EGCG** (Epigallocatechin-3-gallate), **SOD1** (Superoxide Dismutase 1), **TDP-43** (TAR DNA-binding protein 43), **RRM** (RNA Recognition Motif), **Nrf2** (Nuclear factor erythroid 2–related factor 2), **APG** (Apigenin), **ALDH1A2** (Aldehyde Dehydrogenase 1 Family Member A2), **SIRT1** (Sirtuin 1), **HO-1** (Heme Oxygenase 1), **MeHg** (Methylmercury), **MD simulation** (Molecular Dynamics Simulation), **ITC** (Isothermal Titration Calorimetry), **CIU** (Collision-Induced Unfolding), **ESI-MS/MS** (Electrospray Ionization Tandem Mass Spectrometry), **ESI-IM-MS** (Electrospray Ionization Ion Mobility Mass Spectrometry), **ROCK2** (Rho-Associated Protein Kinase 2), **JNK** (c-Jun N-terminal kinase), **p38MAPK** (p38 Mitogen-Activated Protein Kinase), **RT-PCR** (Reverse Transcription Polymerase Chain Reaction), **I113T** (Isoleucine to Threonine mutation at position 113), **A4V** (Alanine to Valine mutation at position 4)

**Table 6:** Future-Oriented Approaches for Enhancing Flavonoid Efficacy in Neurodegenerative Diseases

Title	Description	Technologies/methods	Key references
**Advances in Drug Delivery Systems**	To overcome poor solubility and bioavailability of flavonoids, advanced drug delivery systems have been developed.	Liposomes, SLNs, Polymeric nanoparticles, Nanoemulsions, Micellar systems, Cyclodextrins, Nanocrystals	(L.Chen et al., 2019; Maan et al., 2020; Mattioli et al., 2020; Naeem et al., 2022; Teng et al., 2023)
**Synthetic Analogues and Structural Modifications**	Chemical modifications improve solubility, stability, and bioavailability while adding new biological activities.	Glycosylation, Methylation, Acetylation, Sulfation; Combined with nanocarrier systems	(Teng and Chen, 2019; J. Zhao et al., 2019; Ji et al., 2020; L. Chen et al., 2022)
**Multi-target Therapeutic Strategies**	Flavonoids exert therapeutic benefits by concurrently influencing various molecular pathways, including the inhibition of oxidative stress, the attenuation of inflammation, and the modulation of cell signaling processes.	Multi-targeted approaches for neurodegenerative diseases	(Leonarduzzi et al., 2010; Barreca et al., 2023)
**Personalized Nutrition and Precision Medicine Approaches**	Individual differences influence flavonoid effects, making personalized strategies more effective.	Microbiota-based metabotypes, individualized treatment plans	(Naeem et al., 2022; Barreca et al., 2023)
